# Benevolent Medical Fund

**Published:** 1840-07

**Authors:** 


BENEVOLENT MEDICAL FUND.

[We have much pleasure in giving more extended publicity to the following report; and we earnestly join in the appeal made by the committee to the members of the Association. The slightest consideration must convince every one of the strong claims of this admirable and unexceptionable charity.]

"The central committee beg to present to the Association the following summary of cases relieved since the formation of the benevolent fund: they feel convinced that it is quite unnecessary to say more in proof of the great good that *might* be done, if their means were more extended, seeing how much they have been able to effect with their present limited funds. If each member would add the small sum of Five Shillings to his annual subscription to the Association, and pay it in at the same time, specifying that it was for the purposes of the benevolent fund, the central committee would be enabled to relieve a vast amount of distress and difficulty occurring to meritorious members of the profession and their families, which they are at present compelled, from the scarcity of the funds at their disposal, to refuse.

1. The first case to which the committee were called upon to administer assistance was that of a general practitioner of many years' standing, and of irreproachable character, but whose circumstances were greatly reduced by a severe illness of between three and four years' duration; he thus found himself unable to advance the requisite money to send his son to London to complete his medical education. To assist him in this desirable and necessary matter, the central committee presented him with Fifty Pounds.

2. The next case was that of a general practitioner, advanced in life, and in very reduced circumstances, who was unable to make up the sum due for his rent. To assist him the committee presented him with Ten Pounds.

3. A gentleman of unimpeachable character, who had practised as a surgeon in New South Wales, fell into bad health, and was obliged to return to this country; his pecuniary means being inadequate to his support, he was enabled by the slight help of Twenty Pounds at one time and Ten at another, from this fund, added to the benevolent aid afforded him by the profession at Bath, to prolong his stay in that city (where he found great advantage from the mineral springs) until such time as there was a prospect of his health being reestablished, and of his being able to return to New South Wales, and work out his own independence.

4. Ten Pounds was added to a subscription to place the orphan son of a surgeon on the foundation in the London Orphan Asylum.

5. Five Pounds was remitted to Norwich to relieve the urgent distress of the aged widow of a medical man, who by the death of her husband was left totally unprovided for and dependent upon the benevolent aid afforded her in that neighbourhood.

6. A highly respectable surgeon, a member of the Association, in the prime of life, and just beginning to have a tolerable practice in his profession, was cut off in a few days by typhus fever, caught in the performance of his professional avocations, leaving a wife and seven children totally unprovided for. Forty Pounds was contributed from this fund to a subscription for their benefit, entered into by some benevolent friends of the deceased.

7. A general practitioner in Somersetshire, with a wife and family, became from misfortune in embarrassed circumstances, and was assisted from this fund to the extent of Ten Pounds.

8. Twenty Pounds was added to a subscription for the wife and family of a medical man, who in a fit of temporary derangement had destroyed himself.

9. Ten Pounds was awarded to the widow and family of a highly respectable general practitioner in Gloucestershire.

10. Five Pounds was contributed to assist an aged surgeon with a wife and child in distressed circumstances.

11. Ten Pounds was presented to a general practitioner in Wales, reduced to extreme distress, whose case was very strongly recommended to the committee.

12. Ten Pounds was contributed to assist in the education of two orphan children of a highly respected general practitioner in Lancashire, who died in the prime of life of fever, and whose wife and two children died shortly afterwards, leaving two other children totally unprovided for. This small contribution the committee hope to be enabled to continue annually for a few years, until these children are enabled to support themselves by their own exertions.

13. This was a case of peculiar hardship. A general practitioner in the country, with a wife and family, from ill success in his profession, contracted debts and was committed to prison. After remaining some time in prison he was released by his creditors on condition that he should recommence practice and pay them by instalments. Fortune began to smile upon him; his practice improved and his prospects were encouraging; but his creditors became impatient and again threatened him. To prevent them from fulfilling their threats, a subscription was raised in the neighbourhood, and 350 pounds was raised towards the payment of his debts. Fifty pounds was however still required to make up the amount, and this sum the committee thought themselves justified in contributing to prevent the ruin of an industrious man and humble member of the profession, on whose exertions a wife and small family were dependent.

14. Five Pounds was remitted to a physician in Ireland who was labouring under temporary embarrassment.

For reasons which must be perfectly obvious the committee cannot bring the situation of the individuals who have partaken of the bounty of the society more particularly before the Association. They beg however to assure the members that the awards have been made in conformity with the principles of the society, and the regulations adopted for its guidance; and although they have had frequently reason to regret the limited means at their disposal, yet they have the satisfaction of knowing that in every instance the aid afforded has been of essential service.

Gentlemen disposed to contribute to this excellent charity, may transmit their donations or subscriptions either to Dr. Hastings, Worcester; along with their subscriptions to the Association; or directly to Dr. Conolly Castleton House, near Cheltenham, the treasurer of the fund."

